# Intestinal morphology and host‑ and system‑associated microbiome dynamics during short‑term fasting and refeeding of Atlantic salmon in recirculating aquaculture systems

**DOI:** 10.1038/s41598-026-42939-5

**Published:** 2026-03-10

**Authors:** Christian Karlsen, Andre Meriac, Elisabeth Ytteborg, Gunhild S. Johansson, Gerrit Timmerhaus, René Alvestad, Chris Noble, Jelena Kolarevic

**Affiliations:** 1 Nofima, Muninbakken 9-13, Breivika, Tromsø, Norway; 2 The Norwegian College of Fishery Science, Faculty of Biosciences, Fisheries and Economics, The Arctic University of Norway, Tromsø, N-9037 Norway

**Keywords:** RAS microbiome, Mucosal surfaces microbiome, Skin, Intestine, Vibrio, Ecology, Ecology, Microbiology

## Abstract

Short-term fasting is common in aquaculture prior to handling events, yet its impact on microbial communities and fish health remains poorly understood. We assessed the effects of a 5-day fasting period followed by refeeding on microbial dynamics in a recirculating aquaculture system (RAS), with RAS communities sampled after 5 days of refeeding and the mucosal skin and intestine microbiomes of post-smolt Atlantic salmon (*Salmo salar*) sampled after 6 days. The biofilter and biofilm bacterial profiles remained relatively stable throughout fasting and refeeding, unlike the water microbiome, which showed the most significant changes during refeeding, consistent with distinct microbial growth strategies under fluctuating nutrient conditions. The skin mucus microbiome shares similarities with trends in the water microbiome. No change in skin histomorphometry was observed during the fasting and refeeding periods. In contrast, distal intestinal tissue structures showed differences during the fasting period, indicating potential effects on the intestinal health of salmon. Notably, fasted fish had a higher relative abundance of *Vibrio* bacteria in their remaining digesta. Neither fasting or subsequent refeeding had any effect upon injury-based operational welfare indicators (OWIs) or serum cortisol levels. The study sheds light on the interactions between short-term fasting, microbial community dynamics, and Atlantic salmon health in RAS.

**Supplementary Information:**

The online version contains supplementary material available at 10.1038/s41598-026-42939-5.

## Introduction

Feed withdrawal is regularly applied as a preparatory practice before numerous farming operations in Atlantic salmon (*Salmo salar*) aquaculture^1^. The aim of feed withdrawal is to reduce metabolism and to empty the intestinal tract in order to avoid the excretion of faecal matter during the latter farming operation, which can lead to poor water quality^1^. Lowering fish metabolism helps the fish to tolerate any stressful conditions they may experience during the operation^2^.

The effects of fasting upon fish health and welfare can vary in relation to the life stage of the fish, their prior and ongoing health status, and the environment the fish are subjected to, as reviewed by Hvas et al. (2024)^1^. For example, fasting adult Atlantic salmon for 14 days had no effect on general stress levels or their coping ability when subjected to handling stress^3^. Later studies have also suggested that 4 weeks^4^ or 8 weeks^5^ of feed withdrawal have negligible effects on fish welfare. Previous studies on fasting have largely focused on growth and metabolic changes associated to liver and blood metabolite responses^2^. More recent studies have integrated the immunological perspective and shown that fasting may be associated with improved fish health parameters of the blood, anti-stress and immune responses which relapse to basal levels upon refeeding^6^. Fasting affects the intestinal transcriptome and microbiome in fish, with changes being rapidly reversed after refeeding^7^.

Previous studies on Atlantic salmon fasting often focus on longer fasting periods^4,5,8^, whereas effects from short-term fasting and subsequent refeeding are relatively less well studied^1^. The gut intestinal tract is the first organ affected by fasting and refeeding, both physically and through dietary nutrient sensing following absorption. The intestinal surface is covered by a mucus layer, as is the fish skin whose mucus layer is secreted from epidermal mucus or goblet cells. Mucus has many functions, and one is to have a protective immune function against pathogens^9^. The mucosal surface is in constant contact with the environment, which is potentially rich in microbes making it a principal site for host-bacteria interactions. Atlantic salmon intestinal and skin microbiomes are well studied and the effect of e.g. rearing systems^10^, and diet^11^ on microbial composition has previously been demonstrated, as have changes in the composition of the fish intestinal microbiome in response to fasting^12^.

Although Atlantic salmon is primarily reared in flow-through systems in hatchery production, there is an increased interest in utilizing recirculating aquaculture systems (RAS). RAS has the potential for a better controlled environment, but there are concerns for negative effects, as RAS operate with a high bioactive environment with an enrichment in microbes that may influence fish health and welfare^13^. In RAS, the microbial communities include biofilter-associated autotrophic nitrifiers and heterotrophic bacteria that degrade organic matter. Heterotrophic bacteria respond rapidly to changes in organic matter availability, competing for oxygen and space with autotrophs, but can also protect fish from pathogens, as reviewed in Blancheton et al., (2013)^13^. Changes in the daily feed load greater than 15% can negatively affect nitrification in the biofilter^14^. In addition, the varying concentrations of organic matter in the system are likely to affect the stability of the microbial community^13,15^.

Although previous studies have primarily examined long-term fasting and fish reared in flow-through systems, much less is known about how short-term fasting affects both host and system associated microbiomes in RAS. Given the distinct microbial ecology and higher microbial loads characteristic of RAS, responses to fasting in this environment may differ from those observed in flow-through systems. Given the limited knowledge of short-term fasting in RAS, the present study aimed to: (i) describe temporal changes in the RAS water quality and microbial community during fasting and refeeding, (ii) characterize corresponding patterns in the skin and distal intestinal microbiomes, (iii) examine associated intestinal and skin histomorphological changes, (iv) examine the effects of fasting and refeeding on serum cortisol levels and some commonly used injury-based Operational Welfare Indicators (OWIs) for Atlantic salmon.

## Materials and methods

### Ethical

Handling of fish in this study followed the requirements of the European 2010/63/EU Directive on the protection of animals used for scientific purposes and the experiment was approved by the Norwegian Food Safety Authority (FOTS ID: 29322). All scientific and husbandry personnel in the experiment had documented and updated certification for working with research animals. Both the PREPARE guidelines^16^ and the ARRIVE 2.0 guidelines^17^ were consulted during experimental planning and scientific reporting, respectively.

### Experimental set up

Atlantic salmon parr (20 000 individuals) with an average weight of 10.9 g were produced at Mowi, Bessaker and delivered to the Nofima Centre for Recirculation in Aquaculture (NCRA) in Sunndalsøra^18^ before the start of the experiment. The clinical health status of these fish was audited at the Mowi facility and was consider as good with low mortalities, no signs of disease and 30 fish were negative/free for *Gyrodactylus salaris*. After transport to NCRA, fish were directly placed in 3.3 m^3^ “Cornell-type” dual drain octagonal tanks with flow-through fresh water, which were switched to RAS freshwater, at 12 °C, after 35 days. Smolts were then produced under these RAS conditions using a winter signal (6 weeks with 12 h of light and 12 h of darkness) after which fish were kept of continuous light (24 h) to promote smoltification. After smoltification (29 days before the start of this study), fish were an average weight of 94.0 ± 2.3 g. A subset of these fish was redistributed into six octagonal 3.3 m^3^ tanks, with stocking numbers ranging from 617 to 639 individuals per tank and a stocking density of 17.9 ± 0.5 kg/m^3^. Temperature remained the same (12 °C) and water salinity was increased from 0 ppt to 12 ppt over 24 h. Brackish water (12 ppt) was obtained by mixing approximately one-third of make-up seawater from 40 m depth and two-thirds of make-up freshwater from freshwater wells.

Two identical semi-commercial RAS were used during this experiment. Water treatment consisted of a microscreen belt filter (Salsnes SFK 400, mesh size 120 μm), three chamber moving bed biofilters with Biofilm Chip P (900 m^2^/m^3^ area) biomedia and a forced-ventilated cascade aeration column with counter current temperature-controlled air supply. Water was oxygenated by downflow bubble contactors prior to its return to the tanks. Biofilters in both systems were started at 216 days before the start of the experiment in brackish water (12 ppt) with the addition of chemicals according to Navada et al. (2020)^19^. After 40 days (176 days before the start of the experiment) water salinity was reduced to below 1 ppt until fish were introduced into the systems. To ensure similar RAS water quality, water and biomedia were mixed between systems prior to the start of the experiment. During the experiment, both systems were operated at the following set points: 24 h light, a water temperature of 12 °C, a salinity of 12 ppt, oxygen above 85% saturation in all fish tanks and a pH of 7.8. Fish were fed a commercial Atlantic salmon diet designed for use in RAS (Nutra RC, Skretting, Norway) according to their size and expected growth, using automatic EX04 182 feeders (Poro AB, Kåge, Sweden). Pellet size was changed from 3 mm to 4 mm as the experiment progressed. Feed was delivered continuously (24 h) throughout the experiment, apart from during fasting, which was conducted in one of the RAS, after which feed was reintroduced to the system.

### Sampling timeline

Two parallel semi‑commercial RAS; one control RAS and one fasting RAS, each consisting of three tanks, were used in this experiment. Sampling was conducted at three predefined time points: (i) before the fasting period, (ii) after a 5‑day fasting period, and (iii) after a subsequent refeeding period. Using one fasted and one continuously fed RAS lacks true system-level replication, as all water, biofilm, biomedia, and fish samples are subsamples within a single unit. Consequently, comparisons should only be regarded as descriptive patterns over time and not as treatment effects.

For logistical reasons, RAS and fish samples were collected on separate days. At the first sampling point, RAS samples were collected one day prior to fish sampling and designated D-1 (RAS) and D0 (fish). Immediately after the D0 fish sampling, feed was withheld for five days. Fish were sampled at D5, and corresponding RAS samples collected the following day (D6). After RAS sampling at D6, feeding was resumed. Following six days of refeeding, fish were sampled again at D12, with RAS samples collected the preceding day (D11). When RAS and fish microbiome data are presented together, fish sampling days are used for consistency.

### RAS sampling

Water quality measurements and samples were taken at the tank outlets (*n* = 3 per RAS). Analyses of total ammonia nitrogen (TAN), nitrite and nitrate nitrogen (NO_2_-N and NO_3_-N) and total inorganic carbon (TIC) were conducted as described in Terjesen et al. (2013)^18^. Turbidity was measured with Turbiquant 1500 IR (Merck, Darmstadt, Germany) and alkalinity was measured according to American Public Health Organization^20^. Total suspended solids (TSS) were analysed according to standardized method 2,540 D (TSS dried at 103–105 °C)^20^. Conductivity and salinity were measured using Multi 3410 m and TetraCon^®^ 925–3 conductivity probe (WTW GmbH, Weilheim, Germany). pH was measured using Multi 3410 m and SenTix 940 pH probe (WTW GmbH, Weilheim, Germany). Temperature and oxygen were measured using Polaris portable dissolved oxygen meter (DO, OxyGuard, Denmark).

For microbiome RAS analysis, only samples from the fasted system and the three associated tanks were used. Water samples were collected from three distinct locations within the system: the inlet and outlet of the moving bed biofilm reactor (MBBR), the sump, and outlet of tanks. In addition, samples were taken from the freshwater makeup and seawater makeup sources. At each sampling site, water samples of 500 mL were collected in sterile containers and immediately filtered through a 3 μm filter (Whatman^®^ nitrocellulose filter discs) to get bacteria associated with particles, followed by filtration through a 0.22 μm filter (Mixed cellulose ester membranes, sterile, Whatman™). Biofilms were collected by swabbing using flocked sterile swabs (FLOQSwabs^®^, Copan) at the same sites. Biofilter disks were secured at each timepoint as were the feed pellets used during the experiment. All samples were stored in ZymoBIOMICS Lysis Solution (Zymo Research, Irvine, CA, United States) at −20 °C prior to DNA extraction.

### Fish sampling

Atlantic salmon were netted and immediately anesthetized using an immersion bath with Metomidate (5 mg/L), followed by euthanasia with MS222 (250 mg/L), carried out individually in small buckets. The skin mucus (*n* = 5 per tank per time point on D0, D5 and D12) was accumulated by using the sterile back side of a scalpel swiping from the head and towards the middle of the fish below the dorsal fin. While swiping, a 1 mL pipet was used to aspirate the accumulating skin mucus into Zymo Research Bashing Bead 1.5 mL tubes containing ZymoBIOMICS Lysis Solution. Fish were visually examined for external injuries (*n* = 30 euthanised fish per tank per time point on D0, D5 and D12), scoring the eye, snout, jaw, gill, skin, and fin condition according to 4 levels (0–3): none, minor, moderate, and clear evidence of damage^21^. These 30 fish were measured (length, weight) and the condition factor (*K*) was calculated by the formula *K* = weight (g) ∗ 100/(length (cm))^3^. One piece of skin was aseptically removed by a scalpel from the left side of each fish in the area posterior of the dorsal fin and above the lateral line and placed into 20 mL pots containing 10% buffered formalin (CellStor™ pots, CellPath) and stored at 4 °C. A blood sample was taken by caudal puncture utilizing serum tubes (BD Vacutainer^®^). Tubes stored at 4 °C were centrifuged at 5000 g for 10 min, serum was aspirated and stored at − 20 °C. The serum cortisol concentrations were measured by the enzyme-linked immunosorbent assay (ELISA) kit (Demeditec Diagnostics GmbH) following manufacturer’s instructions. The abdominal cavity was opened and the whole intestine was then aseptically removed. A single-use sterile scalpel blade was then used to make an incision to open the distal intestinal compartment. The gut specimens were then cut lengthways to reveal the inner wall and their digesta removed. The distal digesta consistency was scored to compare digestive status based on visual properties^22^. The system describes digesta with varying degrees of water content. Score 1 describes solid faeces that holds its shape, score 2 describes soft faeces, and score 3 describes faeces with a high liquid content. Score 4 describes cast and score 5 describes an empty intestine^22^. Digesta collection was performed using a new scalpel blade to collect bulk faeces excluding the intestinal mucus layer. Intestinal mucus was collected by swabbing the upper half of the distal intestine. The intestinal microbiome samples were stored individually in bead tubes with lysis solution as described above. The untouched middle part of the distal intestine was placed in formalin as described above.

### Microbiome sample preparation and processing

DNA was extracted from ~ 100 mg feed pellets, distal intestinal digesta and mucus, skin mucus, filtrated water, swabs of biofilm and biofilter. All sample types were processed in Zymo Research Bashing Beads tubes and homogenized with the mechanical bead beater device Precellys^®^24 (Bertin Technologies) for 2 × 20 s at 5000 rpm. The DNA was extracted using ZymoBIOMICS^®^−96 MagBead DNA Kit (Zymo Research, Irvine, CA) according to the manufacturer’s specification. For the low microbial biomass skin mucus samples, the ZymoBIOMICS^®^ DNA Microprep Kit (Zymo Research, Irvine, CA) was used. The extracted DNA samples analyzed by 16S rRNA gene amplicon sequencing of the variable region V3-V4 using custom-designed primers of the commercially available ZymoBIOMICS^®^ Targeted Sequencing Service (Zymo Research, Irvine, CA). The final library was sequenced on Illumina^®^ MiSeq™ with a v3 reagent kit (600 cycles). The sequencing was performed with 10% PhiX spike-in. In addition to the biological samples, the ZymoBIOMICS^®^ Microbial Community Standard was used as positive control; a blank extraction sample was used as a negative control. Quantitative real-time PCR was run on samples with the same primers used in the targeted library preparation. The standard curve was made with plasmid DNA containing one copy of the 16S gene prepared in 10-fold serial dilutions.

Bioinformatic analyses were performed by the ZymoBIOMICS standardized bioinformatics pipeline. Briefly, amplicon sequence variants were inferred from raw reads with potential sequencing errors and chimeric sequences removed using the DADA2 pipeline^23^. Taxonomic classification was performed using Uclust against the curated Zymo Research Database; subsequently, alpha- and beta-diversity metrics were calculated with QIIME v.1.9.1^24^. PCoA plots of the Bray–Curtis–based beta‑diversity analysis and Taxa2ASV Decomposer outputs were generated by the bioinformatics pipeline. Differentially abundant taxa were identified by LEfSe^25^.

Raw sequence data have been deposited in the European Nucleotide Archive (ENA) under accession number PRJEB101828.

### Histology

Formalin fixed skin and distal intestine samples followed standard histological methods with sectioning and staining with Alcian Blue Periodic acid–Schiff (AB-PAS) by the Norwegian Veterinary Institute laboratory (Harstad, Norway) as previously described^26^. The distal intestinal tissue was manually evaluated for 15 characteristic morphological structures adapted from two scoring systems^27,28^. The studied categories were: vacuolization of the enterocytes, large vacuoles in the enterocytes, the thickness of lamina propria (LP) and submucosa (SM), the length and complexity of mucosal folds, intraepithelial lymphocytes (IEL) levels, complex fold oedema, pycnotic cell nuclei, karyhorrexis, rodlet cells, mucosal fold invagination, inflammation (based on the degree of inflammatory cell and lymphocyte infiltration in the LP and SM), lingering mucous, acellular inflammation in muscularis, and calcium deposits. The different morphological characteristics were graded using a 1–5 scoring system for degree of changes, where 1 = normal characteristics/No changes; 2 = mild changes; 3 = moderate changes; 4 = distinct changes, and 5 = severe changes. The histological evaluation was conducted blind.

### Statistical analyses

Water quality parameters were analyzed using repeated measures (RM) ANOVA, using the R v4.5.1 (R Core Team, 2025) packages afex (v1.5-1) and emmeans (v2.0.1). The RM-ANOVA sphericity assumption was checked using Mauchly’s test, and Greenhouse-Geisser corrections were applied where the assumption was violated (*p* < 0.05). Inferential statistics on bioinformatic output data and statistical analysis of fish tissue data were performed using JMP^®^Pro v13.1.0 (SAS Institute Inc. software) or GraphPad Prism v 10.2 (GraphPad Software). Data were assessed for normal distribution using normal quantile plots and evaluated by the Shapiro-Wilk normality test. For comparisons of normally distributed data, group means were tested by one or two-way ANOVA with post hoc pairwise multiple comparisons using Tukey honest significant difference (HSD). Data that were not normally distributed, including welfare scoring, were analyzed by the Kruskal–Wallis test followed by Dunn post hoc tests for all pairs by ranking to compare the groups (*p* < 0.05). The relative abundance of the 50 most abundant taxa was compared across groups by calculating the Spearman correlation. Values *p* < 0.05 were considered significant. To make the visual style more uniform, graphical representations of the processed data were produced in GraphPad Prism.

## Results

### Water quality, fish growth, performance and injury-based OWIs

Since feeding status affects nutrient inputs, microbial activity, and waste production in RAS, water-quality parameters offer context for interpreting how feeding status may affect both microbiome dynamics and system functioning. Parameters related to carbon availability, oxygen balance, turbidity, and nitrogen transformation are particularly relevant. As triplicate tanks within each treatment were maintained within a single RAS, observed differences between the fasted and control systems should be regarded as descriptive patterns. During this experiment, the water quality parameters of the control RAS and the fasted RAS are presented in Table 1, with the results from the repeated measures ANOVA shown in Table 2 and the post hoc comparisons in Tables 3 and 4. Although values varied, no significant differences in temperature (11.7–12.7 °C), conductivity (20.6–21.7 mS/cm), and salinity (12–12.7 ppt) were detected between sampling days or treatments. Across the study period, we observed changes in TIC, DO, turbidity, and pH that co-occurred with the fasting and refeeding periods. TIC varied between systems and sampling points, indicating shifts in carbon cycling co-occurring with feeding status. DO levels changed over time, likely reflecting changes in fish metabolism and microbial activity. Turbidity was significantly influenced by time and also differed between systems, with a marked increase in turbidity in the fasted RAS over the study period. The control and fasted systems showed differences in turbidity at all time points, suggesting disparities in particulate buildup or microbial growth between the systems. Additionally, pH levels increased on day 6 (D6) in both systems. These fluctuations could reflect biological responses, such as changes in fish metabolism and microbial activity, as well as system dynamics, such as biofilter performance and water mixing. No significant changes were observed in total suspended solids (TSS), alkalinity, total ammonia nitrogen (TAN), or nitrite (NO₂-N), indicating stable nitrogen processing. However, nitrate (NO₃-N) increased over time in both systems, consistent with ongoing nitrification.

**Table 1:** Water quality parameters measured at the tank outlet level (*n* = 3, per RAS) one day before fasting (D-1), after the fasting period (D6) and after refeeding (D11) in control RAS and fasted RAS. TIC, Total inorganic carbon; TAN, Total Ammonia Nitrogen; NO_2_-N, Nitrite-nitrogen; NO_3_-N, Nitrate-nitrogen; TSS, Total Suspended Solids.

Water quality parameter	Unit	Sampling day (Mean ± SD)		
		D-1	D6	D11
Temperature (°C)	Control RAS	11.7 ± 0.0	11.8 ± 0.0	12.7 ± 0.0
	Fasted RAS	11.7 ± 0.0	11.7 ± 0.0	12.5 ± 0.0
Salinity (ppt)	Control RAS	12.0 ± 0.0	12.7 ± 0.0	12.3 ± 0.0
	Fasted RAS	12.4 ± 0.0	12.7 ± 0.0	12.1 ± 0.0
Conductivity (mS/cm)	Control RAS	20.6 ± 0.0	21.6 ± 0.0	21.0 ± 0.0
	Fasted RAS	21.1 ± 0.0	21.7 ± 0.0	20.7 ± 0.0
Dissolved oxygen (%)	Control RAS	87.0 ± 2.6	89.3 ± 2.5	86.3 ± 1.5
	Fasted RAS	90.0 ± 2.6	93.0 ± 6.2	87.0 ± 3.5
TIC (mg/L)	Control RAS	23.9 ± 0.1	22.7 ± 0.3	27.7 ± 0.8
	Fasted RAS	23.8 ± 0.4	24.4 ± 0.2	28.0 ± 1.3
Alkalinity (mg CaCO_3_/L)	Control RAS	83.6 ± 2.1	82.7 ± 1.2	88.7 ± 4.6
	Fasted RAS	80.7 ± 1.2	84.7 ± 3.1	88.0 ± 2.0
pH	Control RAS	7.8 ± 0.0	7.9 ± 0.0	7.8 ± 0.0
	Fasted RAS	7.8 ± 0.0	8.0 ± 0.0	7.8 ± 0.0
TAN (mg/L)	Control RAS	0.2 ± 0.0	0.2 ± 0.0	0.2 ± 0.0
	Fasted RAS	0.3 ± 0.1	0.1 ± 0.0	0.2 ± 0.0
NO_2_-N (mg/L)	Control RAS	0.5 ± 0.7	0.1 ± 0.0	0.1 ± 0.0
	Fasted RAS	0.1 ± 0.0	0.1 ± 0.1	0.1 ± 0.0
NO_3_-N (mg/L)	Control RAS	38.7 ± 1.5	50.6 ± 1.6	55.7 ± 8.8
	Fasted RAS	35.9 ± 1.4	41.1 ± 0.6	50.1 ± 10.3
TSS (mg/L)	Control RAS	4.2 ± 0.4	4.0 ± 1.3	4.8 ± 2.1
	Fasted RAS	4.1 ± 0.6	6.2 ± 1.6	6.2 ± 3.9
Turbidity (NTU)	Control RAS	0.8 ± 0.1	0.7 ± 0.1	0.7 ± 0.1
	Fasted RAS	1.1 ± 0.1	1.1 ± 0.0	3.2 ± 0.1

**Table 2:** Repeated measures ANOVA on water quality parameters, showing p-values, F-statistics with degrees of freedom, and effect sizes as partial η^2^. The recorded temperatures, salinities, and conductivities did not have sufficient variance within the fasting or control groups to conduct statistical tests. TIC, Total inorganic carbon; TAN, Total Ammonia Nitrogen; NO_2_-N, Nitrite-nitrogen; NO_3_-N, Nitrate-nitrogen; TSS, Total Suspended Solids.

Dependent variable	Case	*p*	F [df_IV_, df_Error_]	Partial η^2^
Dissolved oxygen (%)	Sampling day	0.04	5.22 [2, 8]	0.57
	Sampling day × Treatment	0.56	0.63 [2, 8]	0.14
	Treatment	0.36	1.07 [1, 4]	0.21
TIC (mg/L)	Sampling day	< 0.001	72.35 [2, 8]	0.95
	Sampling day × Treatment	0.11	3.00 [2, 8]	0.43
	Treatment	0.10	4.58 [1, 4]	0.53
Alkalinity (mg CaCO_3_/L)	Sampling day	0.16	7.21 [2, 8]	0.64
	Sampling day × Treatment	0.40	1.03 [2, 8]	0.21
	Treatment	0.58	0.35 [1, 4]	0.08
pH	Sampling day	< 0.001	256.75 [2, 8]	0.99
	Sampling day × Treatment	< 0.001	77.81 [2, 8]	0.95
	Treatment	< 0.001	1965 [1, 4]	0.99
TAN (mg/L)^a^	Sampling day	0.03	10.94 [1.02, 4.09]	0.73
	Sampling day × Treatment	0.12	3.99 [1.02, 4.09]	0.50
	Treatment	0.84	0.05 [1, 4]	0.01
NO_2_-N (mg/L)^a^	Sampling day	0.40	0.89 [1.02, 4.07]	0.18
	Sampling day × Treatment	0.35	1.11 [1.02, 4.07]	0.22
	Treatment	0.43	0.78 [1, 4]	0.16
NO_3_-N (mg/L)^a^	Sampling day	0.02	13.21 [1.02, 4.09]	0.77
	Sampling day × Treatment	0.48	0.63 [1.02, 4.09]	0.14
	Treatment	0.11	4.06 [1, 4]	0.50
TSS (mg/L)	Sampling day	0.48	0.81 [2, 8]	0.17
	Sampling day × Treatment	0.59	0.57 [2, 8]	0.13
	Treatment	0.33	1.22 [1, 4]	0.23
Turbidity (NTU)	Sampling day	< 0.001	1568 [2, 8]	0.99
	Sampling day × Treatment	< 0.001	1558 [2, 8]	0.99
	Treatment	< 0.001	707 [1, 4]	0.99

^a^ Sphericity assumption violated; reported p-values are Greenhouse-Geisser corrected.

**Table 3:** Post hoc comparisons by sampling day, with Bonferroni corrected p-values. SE, Standard error of the mean; TIC, Total inorganic carbon; TAN, Total Ammonia Nitrogen; NO_3_-N, Nitrate-nitrogen; TSS, Total Suspended Solids.

Dependent variable	Sampling day		Mean Difference	SE	*P*_bonf_
Dissolved oxygen (%)	D-1	D6	−2.67	1.90	0.70
		D11	1.83	1.13	0.54
	D6	D11	4.50	1.00	0.03
TIC	D-1	D6	0.33	0.15	0.28
		D11	−3.98	0.46	< 0.01
	D6	D11	−4.31	0.50	< 0.01
TAN	D-1	D6	0.11	0.03	0.06
		D11	0.04	0.03	0.62
	D6	D11	−0.06	0.00	< 0.001
NO_3_-N	D-1	D6	−8.56	0.62	< 0.001
		D11	−15.58	3.48	0.03
	D6	D11	−7.02	3.90	0.44
pH	D-1	D6	−0.14	0.00	< 0.001
		D11	−0.04	0.01	0.04
	D6	D11	0.10	0.01	< 0.001
Turbidity	D-1	D6	0.06	0.03	0.30
		D11	−1.03	0.02	< 0.001
	D6	D11	−1.09	0.01	< 0.001

**Table 4:** Post hoc comparisons by interaction between treatment and sampling day, with Bonferroni corrected p-values. SE, Standard error of the mean.

Dependent variable	Conditional on sampling day			Mean difference	SE	*P*_bonf_
pH	D-1	Control	Fasting	0.00	0.01	0.52
	D6		Fasting	−0.14	0.00	< 0.001
	D11		Fasting	−0.00	0.01	0.98
Turbidity	D-1	Control	Fasting	−0.37	0.05	0.001
	D6		Fasting	−0.42	0.04	< 0.001
	D11		Fasting	−2.50	0.06	< 0.001

Conditional on treatment						
pH	Control	D-1	D6	−0.07	0.00	< 0.001
			D11	−0.03	0.01	0.13
		D6	D11	0.04	0.01	0.07
	Fasting	D-1	D6	−0.21	0.00	< 0.001
			D11	−0.04	0.01	0.09
		D6	D11	0.17	0.01	< 0.001
Turbidity	Control	D-1	D6	0.08	0.04	0.26
			D11	0.04	0.03	0.96
		D6	D11	−0.04	0.02	0.20
	Fasting	D-1	D6	0.03	0.04	1.00
			D11	−2.10	0.03	< 0.001
		D6	D11	−2.13	0.02	< 0.001

Water quality parameters of the makeup freshwater and seawater for the RAS during the experimental period, from D-1 to D11, are presented in the Supplementary Information Table S1. Fish (*n* = 15 per group) and organ measurements of the sampled fish (Supplementary Information Table S2) showed that weight increased during the experimental period, with a lower condition factor observed in the fasted group at the end of the fasting period (D5). Welfare scoring of the eye, snout, jaw, gill, skin and fin condition (*n* = 90 per group per time point) demonstrated no differences between systems or over time (Supplementary Information Figure S1).

### Impacts of fasting on system microbiota

In this study, we investigated biomedia, water (filtered through 3 μm and 0.2 μm filters) and biofilm from different parts of the RAS including the mucosal barriers of post-smolt Atlantic salmon. These samples were grouped into skin mucus, distal intestinal mucus, and distal intestinal digesta. Analysis of 16S rRNA gene sequencing data from system and fish tissue samples revealed temporal differences that coincided with the fasting period and the subsequent refeeding period on both the system and the fish. The microbiome residing in fish tissues exists in continuity with that of the surrounding system environment, which overlap to a varying degree. In addition, we had samples from the distal intestinal mucus and digesta from fish in the continuously fed control system at the same three different timepoints; before the start of fasting D0, after a 5-day fasting period D5, and after the refeeding period (D12). A total of 16,981,682 reads were retained for subsequent statistical analysis among the 340 samples in the dataset, with a mean ± SD of 49.946 ± 15.475 per sample.

**Fig. 1:**
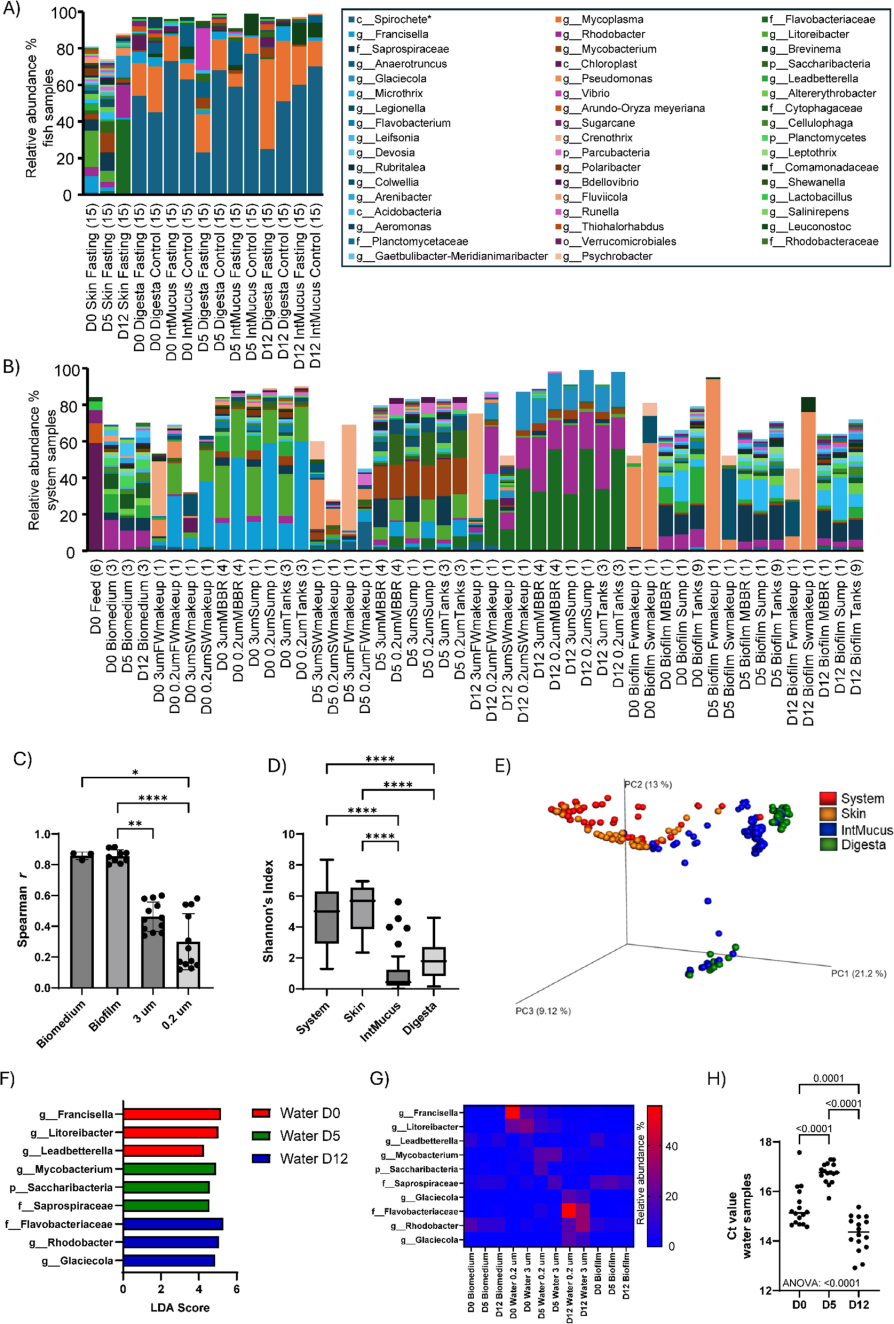
Effect of fasting and refeeding on microbial community composition. Taxonomic barplot containing the 50 top dominating genera (highlighted in boxed area) for the whole sample set, in fish samples (**A**) and the fasted RAS (**B**). The numbers in brackets after group names represent sample size. (**C**) Spearman correlation analysis of the dominating genera between day 0 (D0), D5 and D12 by biomedium (*n* = 3), biofilms (*n* = 9) and water filtrates of 3 μm (*n* = 12) and 0.2 μm (*n* = 12) in the fasted system. (**D**) Box-plots corresponding to the Shannon index (alpha diversity). (**E**) Principal coordinates analysis corresponding to the Bray–Curtis dissimilarity index (beta diversity). (**F**) To identify the taxonomic biomarkers of water a linear discriminant effect size (LEfSe) analysis was performed on all three timepoints with 3 μm and 0.2 μm combined from the makeup water, the moving bed biofilm reactor (MBBR), the system’s sump, and tank outlet water. The three top taxa identified using linear discriminant analysis (LDA) score per time point are presented. (**G**) The relative abundance of the water biomarkers by system groups (biomedium, biofilm from swabs and water (3 μm and 0.2 μm). (**H**) Ct-values were obtained from tank samples (*n* = 3), MBBR samples (*n* = 4), and sump samples (*n* = 1) collected on 3 μm and 0.2 μm filters, totalling 16 samples per timepoint. Significant differences between groups in B and C determined by pairwise testing, performed using Kruskal-Wallis and Dunn´s multiple comparison tests: *p < 0.05, **p < 0.01, ***p < 0.001, ****p < 0.0001. In G, an ANOVA coupled with a Tukey’s HSD post-hoc test for pairwise comparisons was performed. c_Spirochete* as determined by Blastn of dominant amplicon sequence variant (ASV). Abbreviations: FWmakeup, fresh water to maintain system volume; SWmakeup, seawater to maintain system volume; MBBR, Moving Bed Biofilm Reactor; sump, system’s low point before pumping; 3 μm and 02 μm, water filtered through 3 μm and 0.2 μm filters; IntMucus, distal intestinal mucus.

Using the top 50 dominating identified taxa across all samples, the microbial community profiles for fish samples are shown in Fig. 1A and for system samples in Fig. 1B. There was an observable difference in relative abundance between system and fish and to a varying degree between timepoints. In biomedium, the dominant genus was the *Rhodobacter* (12%). In water, the dominant relative abundance of dominant taxa changed between time points and was at the genus level dominated by *Francisella* (D0 3 μm and 0.2 μm: 15.8/55.5%, D5 3 μm and 0.2 μm: 1.8/8.5%, D12 3 μm and 0.2 μm: 0/0%), *Litoreibacter* (D0 3 μm and 0.2 μm: 27.3/22.0%, D5 3 μm and 0.2 μm: 8.3/12.0%, D12 3 μm and 0.2 μm: 1.0/1.8%), *Mycobacterium* (D0 3 μm and 0.2 μm: 4.3/1.3%, D5 3 μm and 0.2 μm: 19.3/18.8%, D12 3 μm and 0.2 μm: 4.5/3.5%), *Rhodobacter* (D0 3 μm and 0.2 μm: 3.0/0%, D5 3 μm and 0.2 μm: 2.5/0.5%, D12 3 μm and 0.2 μm: 32.3/17.5%), *Glaciecola* (D0 3 μm and 0.2 μm: 0/0%, D5 3 μm and 0.2 μm: 0/0%, D12 3 μm and 0.2 μm: 12.8/18.5%). Biofilms of the system were dominated by *Microthrix* (D0/D5/D12: 9.0/11.7/12.7%) and *Leadbetterella* (D0/D5/D12: 11.7/1.7/3.0%). The intestine was dominated by *Spirochete* (D0/D5/D12: 58.8/56.8/51.5%), as identified using Blastn search against the nr/nt NCBI database of the most dominant amplicon sequence variant (ASV) (Supplementary Information Figure S2) and *Mycoplasma* (D0/D5/D12: 16.5/13.5/29.3%).

To evaluate whether relationships between the system and fish microbiomes differed across timepoints spanning fasting and refeeding periods, we compared samples collected before fasting (D0), after the fasting period (D5) and after refeeding (D12). On the system level grouped by biomedium, biofilm and water filtrates of 3 μm and 0.2 μm Spearman correlation using the relative abundance levels of dominating taxa (top 50), showed that correlation values from D0 vs. D5 and D12 and D5 vs. D12 were higher in biomedium and biofilms compared to water (Fig. 1C). Changes in community composition were smaller in biomedium and system biofilms compared to water during the fasting and refeeding periods. Analyzing a total of 240 samples from the fasted RAS and fish samples, we observed a significant lower microbial community diversity in the intestinal mucus and digesta compared to the system and fish skin mucus (Fig. 1D). Principal Coordinate Analysis (PCoA) further supported distinct differences in community composition between the system/fish skin mucus and the intestinal mucus/digesta groups (Fig. 1E). Specific bacterial taxa that were significantly affected by fasting and refeeding in the water were identified using linear discriminant effect size (LEfSe). Differentially abundant taxa were identified using linear discriminant analysis (LDA). Taxa with an LDA score of ≥ 2.0, indicating a meaningful effect size, are presented in Supplementary Information Table S3. The top three affected taxa in the water included *Francisella*,* Litoreibacter*, and *Leadbetterella* on D0, *Mycobacterium*,* Saccharibacteria*, and *Saprospiraceae* on D5, and *Flavobacteriaceae*,* Rhodobacter*, and *Glaciecola* on D12 (Fig. 1F). The relative abundance of these taxa was markedly higher in water samples (both 3 μm and 0.2 μm) than that of biomedium and swabbed biofilms at the same timepoint, although *Leadbetterella*,* Saprospiraceae*, and *Rhodobacter* were also abundant in biomedium and biofilms (Fig. 1G). The change in dominant taxa in water between timepoints, co-occurred with a reduced total microbial bacterial load after the fasting period, as shown with quantitative measures of Ct-values (Fig. 1H).

### Impacts of fasting on the skin and distal intestine

**Fig. 2:**
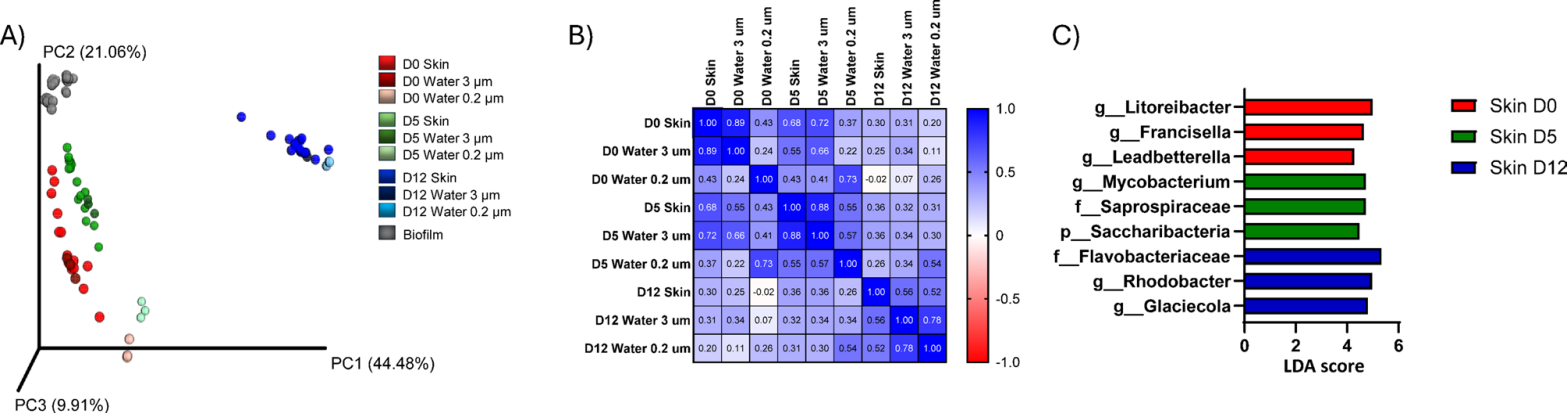
Impacts of fasting on skin mucus. (**A**) Principal coordinates analysis corresponding to the Bray–Curtis dissimilarity index (beta diversity) between tank biofilm, skin mucus, and water (3 μm and 0.2 μm) at the start of fasting D0, after fasting D5 and after refeeding D12. (**B**) Spearman correlation analysis of the top 50 dominating taxa in the dataset between D0, D5 and D12 by skin mucus and tank water filtrates of 3 μm and 0.2 μm in the fasted system. (**C**) To identify the taxonomic biomarkers of skin mucus a linear discriminant effect size (LEfSe) analysis was performed on all three timepoints. The top three taxa identified using linear discriminant analysis (LDA) score per time point are presented.

We next wanted to investigate the effect of fasting and refeeding on the skin mucus microbial communities. Beta diversity analysis of community composition using Bray-Curtis dissimilarity index (Fig. 2A) showed that fasted (D5) and refed (D12) communities separated in comparison to the start of fasting (D0). There was also a distance to the tank biofilms. However, water samples were more similar to skin mucus samples, with 3 μm filtrates being the most similar. The distance to 0.2 μm water samples was greater. The pattern repeated when using Spearman correlation analysis based on the top 50 taxa (Fig. 2B). This suggested that the skin mucus microbiome composition was similar to the water environment at all time points. Strikingly, when LEfSe analysis was performed on the skin groups (skin D0 vs. skin D5 vs. skin D12), the top three bacterial taxa that were deemed discriminative for a particular group (Fig. 2C), were the same taxa identified when performing LEfSe analysis on the respective water groups (water D0 vs. water D5 vs. water D12, Fig. 1F).

**Fig. 3:**
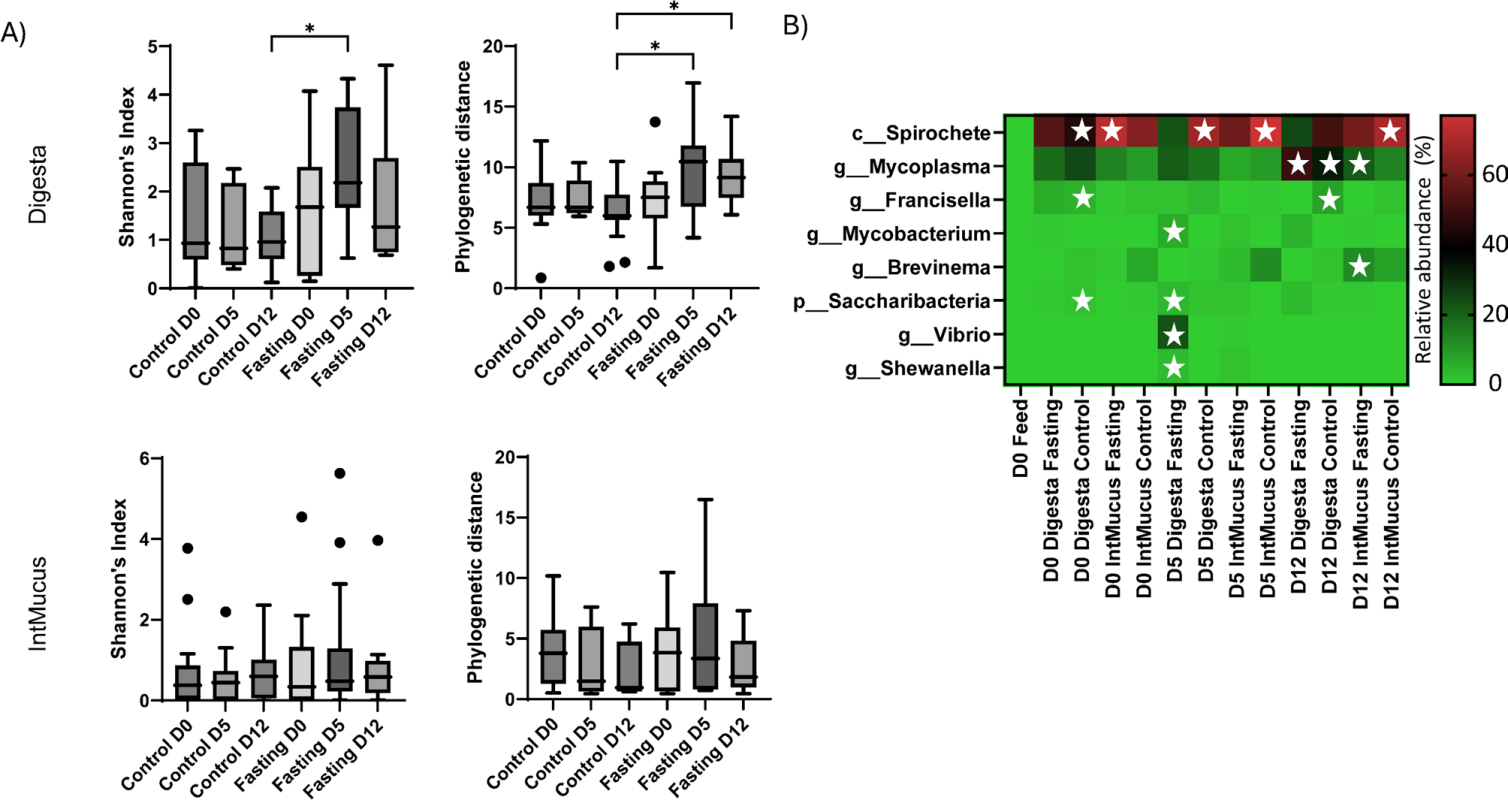
Intestinal diversity and dominant taxa. (**A**) Comparison of alpha diversity (Shannon and Phylogenetic distance) in digesta and intestinal mucus (IntMucus). (**B**) Heatmap of relative abundance of bacterial taxa significant by LEfSe analyzed separately by digesta (control and fasting) and intestinal mucus (IntMucus: control and fasting) vs. feed by timepoints D0, D5 and D12. Stars denote time identified as significant by LEfSe (LDA score > 4, *p* < 0.05).

**Fig. 4:**
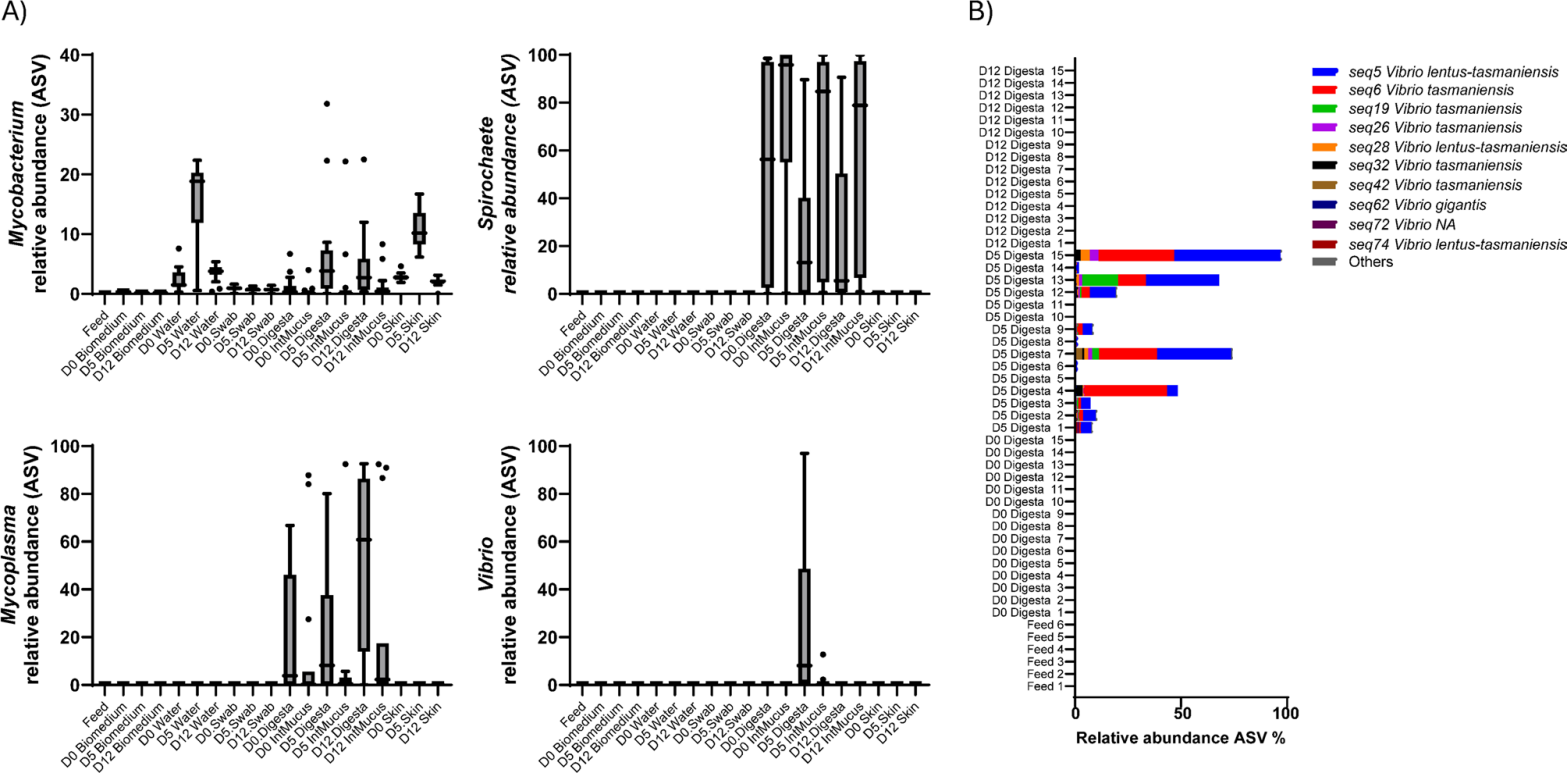
Compositional dynamics of the fasted intestine. (**A**) Relative abundance (ASV) of *Mycobacterium*, *Spirochete*, *Mycoplasma* and *Vibrio* observed in the fasted RAS and fish tissue before fasting (D0), after fasting (D5), and after refeeding (D12) periods. (**B**) Relative abundance % ASVs plot assigned to the genus *Vibrio* in the digesta of before (D0), after fasting (D5) and after (D12) refeeding. Abbreviations: ASV, amplicon sequence variant; IntMucus, intestinal mucus.

Overall, the distal intestinal mucus and digesta microbiomes maintained their diversity level between timepoints in the control and fasted system (Fig. 3A). Separate LEfSe analysis was performed using the three time points D0, D5 and D12 in addition to feed for (i) Digesta of the control system, (ii) Intestinal mucus of the control system, (iii) Digesta of the fasted system, (iv) Intestinal mucus of the fasted system. Bacterial taxa that were deemed discriminative for a particular group with an LDA score > 4 and within the top 50 relative abundance across all samples in the data set included 8 taxa plotted in Fig. 3B. *Spirochete* and *Mycoplasma* were found discriminative in several comparisons. Both *Spirochete* and *Mycoplasma* are dominant in the intestine of fish but were not reflected in the environmental samples of the system (Fig. 4A). This was in contrast to *Mycobacterium*, which was reflected in the environment, especially the water (Fig. 4A). *Vibrio* reached a maximum relative abundance of 97% (mean 23%) in the digesta samples after fasting. This effect of fasting in the digesta of fasted fish was not reflected in the environment, as *Vibrio* was not detected before D5 and only in water (mean 0,039%). In the fasted system, the intestinal microbiome composition changed over time, including a marked increase in the relative abundance of *Vibrio* genus members during fasting (Fig. 4B).

### Histomorphology of the skin and intestine, and serum cortisol profiles

**Fig. 5:**
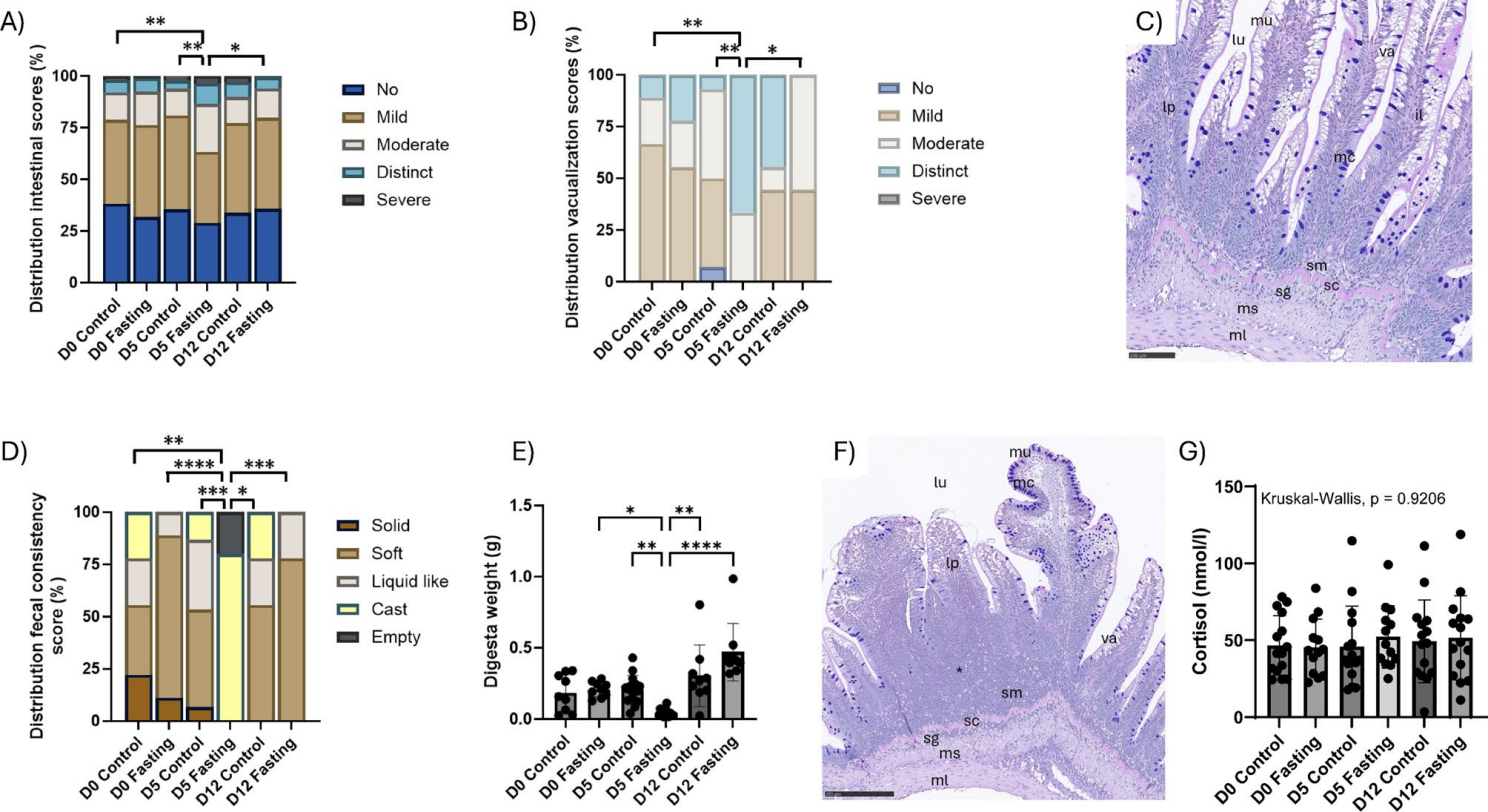
Histomorphometry of the distal intestine and faecal consistency observed in fasted and refed Atlantic salmon (*n* = 9 at D0 and D12, *n* = 15 at D5 per group). (**A**) Overview of combined intestinal score from distal intestine scored from none to severe changes. (**B**) Summary of total score of vacuolization of the enterocytes. (**C**) Example image of a highly vacuolated distal intestine representing the normal condition when fish are fed. (**D**) Distribution of faecal consistency scores. (**E**) Distal intestinal digesta weight. (**F**) Example image of inflammatory (*) reaction in submucosa (sm) and lamina propria (lp). (**G**) Serum levels of cortisol. Significant differences between groups in A, B, D, E and G were determined by pairwise testing, performed using Kruskal-Wallis and Dunn´s multiple comparison tests: *p < 0.05, **p < 0.01, ***p < 0.001, ****p < 0.0001. For simplicity, only differences to group D5 Fasting in F are shown. Abbreviations: il, intraepithelial lymphocytes; lp, lamina propria; lu, lumen; mc, mucous cells; ml, muscularis longitudinalis; mu, mucosa; ms, muscularis sircularis; sc, stratum compactum; sg, stratum granulosum; sm, submucosa; va, vacuolisation.

The skin parameters examined in this trial, shown in Supplementary Figure S3, did not differ significantly between the control and fasting groups. Score distribution of intestinal histomorphometric measurements showed a higher degree of affected tissue after the fasting period in the fasted system (Fig. 5A). The score distribution across the 15 different categories is shown in Supplementary Figure S4, with significant differences after the fasting period detected in the categories vacuolisation of the enterocytes, the thickness of the lamina propria, and the complex fold oedema. Vacuolization of the enterocytes was reduced in fasted fish (Fig. 5B), when compared to a normally fed fish (illustrated in Fig. 5C). There was a marked difference in faecal score profiles from fasted fish (Fig. 5D) with 12 of the 15 fish with casts and the three remaining distal intestines empty of digesta accompanied with the lowest digesta content by weight (Fig. 5E). Intestinal digesta scored as casts were identified in other groups indicating that not all fish ate in a daily manner during the experiment. Enteritis was detected at D0 in both the control and fasting groups. In the fasted RAS, inflammatory changes (illustrated in Fig. 5F) were observed at D0, which then increased at D5, before returning to levels similar to the control RAS after refeeding (Supplementary Figure S4). Serum cortisol levels did not change (Fig. 5G).

Together, these experimental findings provide a basis for interpreting how Atlantic salmon tissues and microbial communities varied during the short‑term fasting and refeeding periods in our RAS study.

## Discussion

The study of microbial communities in relation to environmental changes in RAS offers valuable insights into the resilience and adaptability of microbes within these ecosystems. One factor that is widely recognised to significantly influence microbial communities is the nutritional status of the environment. During the fasting period, bacterial abundance was lower, coinciding with lower TIC and higher DO, indicating reduced microbial activity. After refeeding, the opposite pattern occurred with elevated TIC and decreased DO, which might reflect intensified microbial metabolism and system response. These changes in the nutritional status of the RAS are likely to influence the ecological fitness of taxa. The current experiment showed that abundant taxa were more stable in biomedium and biofilms compared to water in the fasted and refed periods, a pattern characteristic of K-selected taxa, which typically thrive in stable, resource-constrained environments^29,30^. The stability of K-selected taxa in the biomedium and biofilms contrasts with the fluctuating populations in water, reflecting different survival strategies. After fasting, the most abundant taxa in RAS water included *Mycobacterium*, *Sacchribacteria* and *Saprospiraceae*. During the refeeding period, higher relative abundances of *Flavobacteriaceae*, *Rodobacter* and *Glaciecola* were observed. These taxa may have potential roles in organic matter processing.

In our system, *Saprospiraceae* was abundant in biomedia and biofilms. From RAS water, it was associated with 3 μm filtered water, indicating that these bacteria were not part of the planktonic free-living bacteria in the water column as *Mycobacterium* and *Saccharibacteria*, which were associated with 0.2 μm filtrates. These results indicate the importance of understanding microbial behaviour at different particle levels in a RAS. Of note, changes in relative abundance after fasting could be an effect of the reduction in absolute abundance based on 16S rRNA copy numbers detected from water samples, e.g. because of the loss of other taxa. After refeeding, these taxa declined, and new taxa became dominant in the water column.

Similar to prior research that examined various mucosal surfaces, alpha diversity was higher in the skin mucosa compared to the intestine^31,32^. Differential abundance analysis in combination with correlation analysis showed abundance changes for several taxa of the skin mucus, reflecting the same taxa as those identified in water, especially to 3 μm filtrated water. Highly variable host-associated microbial communities have been observed in marine animals under stress, which is suggested to be because the animals cannot regulate their microbiome^33,34^. Although we cannot exclude that fish or the skin experience some level of stress associated with the RAS environment, that makes it more unresponsive to colonization, a more plausible explanation is the high level of bacterial load in the water that carries over to mucus sampling. This observation is further supported by the consistent changes observed in both water and skin mucus at all three investigated time points. Others similarly report an overlap of Atlantic salmon skin-mucus communities with water communities^31,35^. The degree of overlap may be influenced by particle size in the water as beta-diversity analysis separates communities of free-living taxa (0.2 μm) compared to 3 μm particles, from skin in the current trial. Also, a change in mucus properties may account for variations in the diversity of microbiota residing in the mucus of the fish. However, we found no difference in the appearance of skin mucus cells between treatments, which contrasts with a decrease in the number of epidermal mucus cells of short-term fasted Atlantic salmon in a previous study^36^.

This experiment further assessed the relationship between fasting and faecal consistency scores. The increased incidence of higher faecal scores and lower digesta weights in fasted fish suggests that the feed withdrawal time influenced faecal consistency properties. This aligns with previous studies reporting that gut evacuation occurs after 24 h and 48 h in 150–300 g smolts held at 10–14 °C and 6 °C, respectively^37^. However, no significant difference in alpha diversity was observed in the digesta and intestinal mucus between groups, which could be due to the variability between individual fish in this trial. The lack in difference contrasts with the lower richness in high (cast-rich) faecal scores observed in other Atlantic salmon trials^22,38^. It is commonly accepted that diet can affect the composition of the microbial community. However, due to the process of feeding, an individual’s observable microbiome at the time of sampling might be a carryover from the last meal as the digesta can to a varying degree be influenced by microbial DNA from the feed^39^. Additionally, time from feeding is shown to influence both faecal scores and changes in the gut microbiota^38^, although the effect of microbial DNA from the feed was found to be negligible in this trial.

There were significant differences in digesta microbial community composition between fed and fasted fish. The most abundant taxa differing between fed and fasted fish, defined as those identified as significant by LEfSe (Fig. 3B), include a mix of environmentally derived bacteria and bacteria associated with the distal intestine (Fig. 4A). For example, *Saccharibacteria* and *Mycobacterium* are both found in abundance in the surrounding water, and we assume that their presence in the distal intestine is transient in origin. Our analysis identified *Mycoplasma* and Spirochaetes ASVs as part of the distal intestinal microbiome, indicating potential true residents of the digesta and intestinal mucosa.

The high abundance of *Mycoplasma* and the taxa belonging to the Spirochaetes phylum, *Brevinema* and *Spirochete*, have been identified by others as prevalent in the intestine at various life stages with community compositions often dominated by a few taxa^32,39–41^. It has recently been suggested that Atlantic salmon have likely evolved in close association with *Mycoplasma*^42^. Although its function is still being elucidated, it is regarded as highly host-dependent due to its intracellular lifestyle with a positive effect on the salmonid host^42–45^.

Our results provide insights into the specific bacterial lineages that are most sensitive to fasting and refeeding in the Atlantic salmon intestine. Although the intestinal microbial communities subjected to fasting displayed no differences in diversity, an altered composition of main taxa was observed in fasted fish compared to fed controls. Both *Vibrio* and *Shewanella* showed higher relative abundance in the intestine of fasted fish. This increased relative abundance of both taxa was normalized within the refeeding period. Both genera are common taxa of Atlantic salmon digesta^39^. The increase in Vibrionaceae as an effect of fasting is reported in previous fasting studies with fish^7,46^, and in digesta with high (cast-rich) fecal score linked to rising summer temperatures^38^ and heat stress^47^, further supporting the observed relationship in our study. The lack of food is likely to change the nutritional niche within the intestinal digesta. The remnants of digesta in the intestine of fasted fish that resembled pellet (cast) favour taxa which can adapt to changing nutrient conditions. Both *Vibrio* and *Shewanella* are commonly found in marine environments, adapt rapidly to environmental stressors, and utilize various carbon and energy sources efficiently. In times where food is sparce, causing a lower nutritional environment, these taxa may outcompete other bacteria.

We distinguished that *Vibrio* reached a higher relative abundance in the digesta part, which coincided with the fasting period in the fasted RAS. As *Vibrio* spp. can degrade mucus^48,49^, the mucus present in the digesta of fasted fish may act as a nutrient source when separated from the intestinal wall, where immune activity would otherwise inhibit proliferation of the *Vibrio* taxon.

In the zebrafish intestine, the relative abundance of *Vibrio* increases with inflammation^50^ and fasting^7^, supporting a link between fasting-induced microbial shifts and alterations in intestinal gene expression.

Whilst literature on the effects of fasting on the behaviour and injury status of Atlantic salmon is often limited, previous work has shown that feed withdrawal (3 days) leads to increased dorsal fin damage in salmon parr weighing 40 grams^51^. Restrictive daily feed amounts may similarly induce behaviour changes in the form of aggressive interactions that lead to dorsal fin damage in Atlantic salmon parr^52^. However, subjecting post-smolt Atlantic salmon to prolonged feed withdrawal (56 days) did not lead to aggression in another study^5^. In our study on post-smolts we found no evidence of differences in any of the monitored injury-based operational welfare indicators in relation to fasting, including dorsal fin damage. This may be because salmon are surmised to become less aggressive as they transition from the parr to post-smolt stage, meaning life stage is a critical factor when considering how fasting can affect fish welfare (Hvas et al., 2024^1^ and references therein). Physiological stress within the population, which in turn affects immune signals, can also shape the intestinal microbiota^53^. Data on serum cortisol levels did not suggest that fish were more stressed by either fasting, refeeding or the presence of *Vibrio* in the digesta, consistent with *Vibrio* being associated mainly with the digesta than the intestinal mucus layer. It is likely that the increase in relative abundance is driven by a decrease in OTUs from other taxa, rather than an absolute increase.

The relative abundance of *Vibrio* was highest in the digesta, which could suggest that nutrient availability and metabolic function are stronger drivers for *Vibrio* enrichment in the intestine when cast is produced following a fasting period. If this is a generalized response to fish not eating, it could suggest why Vibrionaceae become predominant in the intestinal samples of unhealthy Atlantic salmon in the seawater stage^43,54^. Interestingly, bacterial taxa and the fasting-induced perturbations shown by the histomorphometrics of the intestine were either outcompeted or restored within 6 days after refeeding. This underscores the plasticity in microbial ecology and intestinal physiology in response to fasting and refeeding periods, and aligns with previous reports of rapid histological and enzymatical changes to fasting in the intestine of Atlantic salmon^55,56^.

Our results give insight into the spatial fluctuations in host-associated and environmental microbial community dynamics across a RAS. A limitation of this study was that each treatment condition was represented by only one RAS, which limits findings as descriptive patterns consistent with fasting rather than definitive causal effects. Despite this limitation, the temporal dynamics observed within each system provide valuable insight into how microbial communities and intestinal responses may vary during short-term feed withdrawal and refeeding. Here, we show that short-term fasting and refeeding periods coincided with changes in water microbial communities, which were stronger compared to changes observed in biomedium and system biofilms. During the short period of feed withdrawal, we further observed changes in the morphometrics of the distal intestine and the intestinal digesta microbial community compositions, with higher relative abundance of *Vibrio* in the fasted system. No differences in injury-based OWIs or serum cortisol levels were observed within and between treatments in this study. Future work should further focus on linking changes in the microbiomes to direct measures of host health.

## Supplementary Information

Below is the link to the electronic supplementary material.

Supplementary Material 1

Supplementary Material 2

## Data Availability

All relevant data are within the manuscript and its Supplementary material files. The sequencing data have been deposited in the European Nucleotide Archive (ENA) under accession number PRJEB101828 and will be publicly available at https:/www.ebi.ac.uk/ena/browser/view/PRJEB101828 upon publication.
