# Supplementary material for: Intestinal morphology and host‑ and system‑associated microbiome dynamics during short‑term fasting and refeeding of Atlantic salmon in recirculating aquaculture systems
Source: Sci Rep. 2026 Mar 10;16:12906. doi: 10.1038/s41598-026-42939-5 (PMC13096183; doi:10.1038/s41598-026-42939-5)
Supplement: Supplementary file 2 — Supplementary Material 2 [file 41598_2026_42939_MOESM2_ESM.pdf]

Supplementary Information

Short-term fasting and refeeding are associated with variations in intestinal morphology and microbiome dynamics in Atlantic salmon and recirculating aquaculture systems

Christian Karlsen[1*], Andre Meriac[1], Elisabeth Ytteborg[1], Gunhild S. Johansson[1], Gerrit Timmerhaus[1], René Alvestad[1], Chris Noble[1], Jelena Kolarevic[1,2]

[1] Nofima, Muninbakken 9-13, Breivika, Tromsø, Norway

[2] Present Address: The Norwegian College of Fishery Science, Faculty of Biosciences, Fisheries and Economics, The Arctic University of Norway, N-9037 Tromsø, Norway

* Corresponding author: CK, phone: +4764970391, email: christian.karlsen@nofima.no

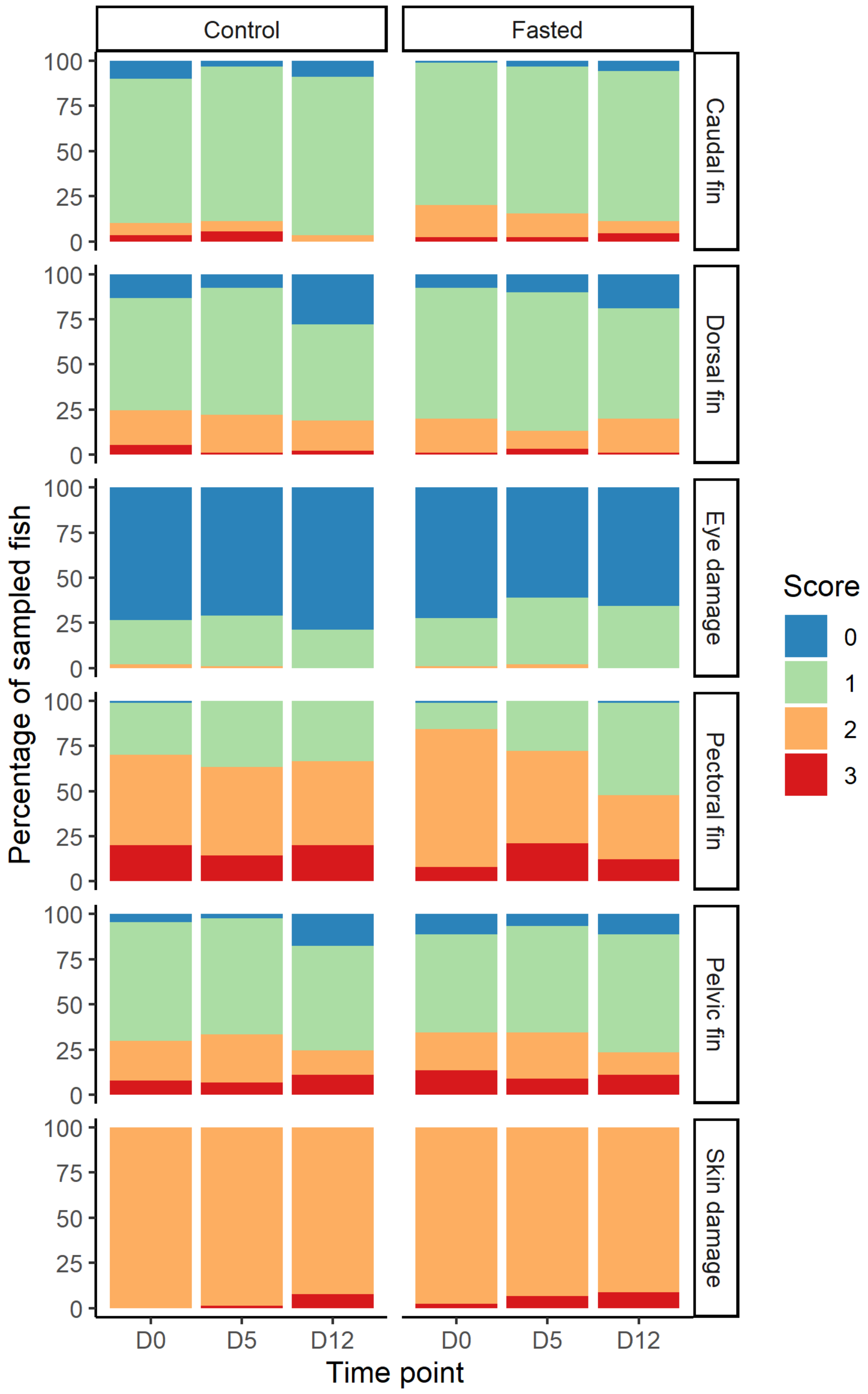

Figure S1 Welfare scoring of fish (total of 540 fish, *n* = 90 per group per time point). Fish were visually examined for scoring the eye, snout, jaw, gill skin and fin condition according to 4 levels (0-3): 0 = none, 1 = minor, 2 = moderate, and 3 = clear evidence of damage.

>seq1

AGCCAAGAATATTCCGCAATGGGGGAAACCCTGACGGAGCGACACTGCGTGAATGAT
GAAGGCCTTCGGGTTGTAAAGTTCTTTTATAAAGGAAGAATAAGTTGGGTAGGAAATG
ATTCAATGATGACGGTACTTTATGAATAAGTCCCGGCTAATTACGTGCCAGCAGCCGCG
GTAATACGTAAGGGACGAGCGTTATTCGGAATTACTGGGCGTAAAGGGCGTGTAGGCG
GCCGATTAGGCTGAGTGTTAAAGACTGGGGCTCAACTCCAGAAAGGCATTCAGAACC
GGTTGGCTAGAATCTGGTGGAAGACAGCGGAATTTCCTGTGTAGCGGTGGAATGCATA
GATATGGGAAGGAACACCAAAGGCGAAGGCAGCTGTCTATGCCGAGATTGACGCTGA
GGCGCGAAAGTGTGGGGATCAAA

Figure S2 Fasta sequence of the dominating ASV assigned to the Spirochaeta by BLAST search using the core nucleotide databases.

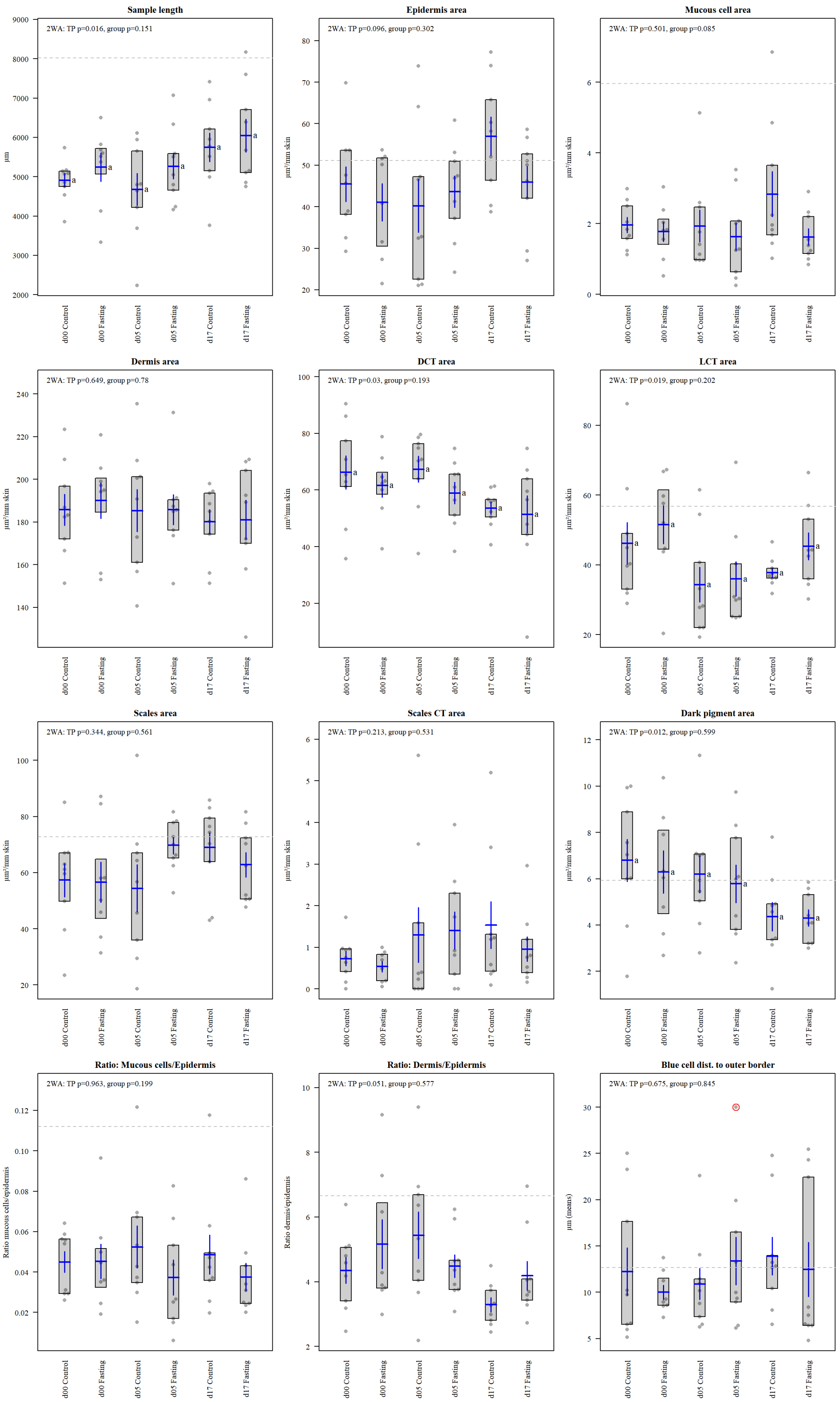
Sample length
2WA: TP p=0.016, group p=0.151
µm
Epidermis area
2WA: TP p=0.096, group p=0.302
µm²/mm skin
Mucous cell area
2WA: TP p=0.501, group p=0.085
µm²/mm skin
Dermis area
2WA: TP p=0.649, group p=0.78
µm²/mm skin
DCT area
2WA: TP p=0.03, group p=0.193
µm²/mm skin
LCT area
2WA: TP p=0.019, group p=0.202
µm²/mm skin
Scales area
2WA: TP p=0.344, group p=0.561
µm²/mm skin
Scales CT area
2WA: TP p=0.213, group p=0.531
µm²/mm skin
Dark pigment area
2WA: TP p=0.012, group p=0.599
µm²/mm skin
Ratio: Mucous cells/Epidermis
2WA: TP p=0.963, group p=0.199
Ratio mucous cells/epidermis
Ratio: Dermis/Epidermis
2WA: TP p=0.051, group p=0.577
Ratio dermis/epidermis
Blue cell dist. to outer border
2WA: TP p=0.675, group p=0.845
µm (means)
d00 Control
d00 Fasting
d05 Control
d05 Fasting
d17 Control
d17 Fasting

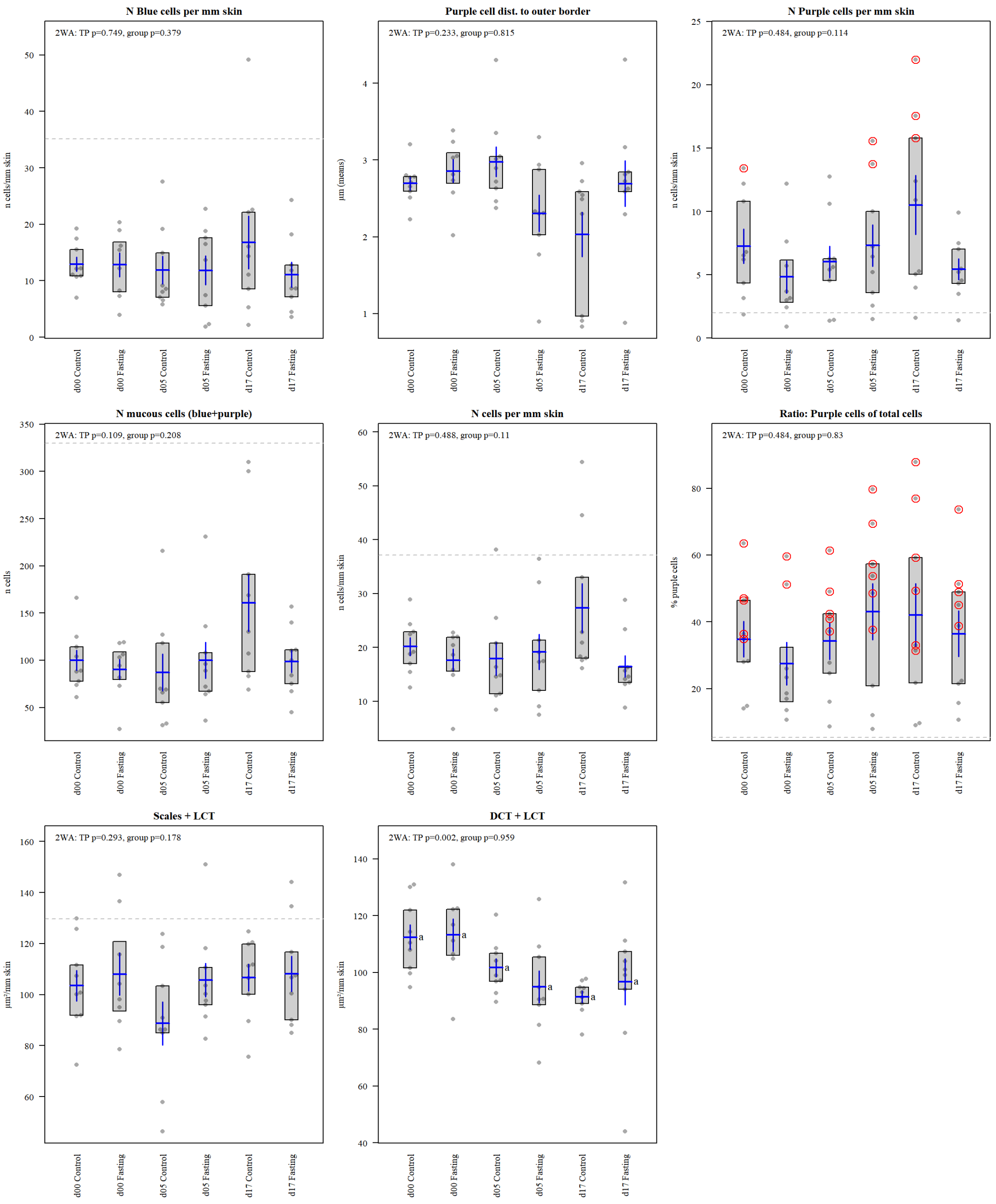

Figure S3 Atlantic salmon skin tissue comparisons (n = 9 per group). Plots illustrate the areas of skin components and variables for mucous cells. The grey boxes represent the 2nd and 3rd quartiles. The blue horizontal lines denote the mean values, and the blue vertical lines indicate the +/- SEM. The grey horizontal dashed lines show the expected median values for the current fish size for each variable. Red circles highlight values that are more than two SDs away from the respective adjusted median values. Results from a two-way ANOVA are displayed above each plot. Compact letter displays indicate statistically distinct groups based on an independent group-wise comparison across all groups. LCT refers to loose connective tissue. DCT refers to dense connective tissue.

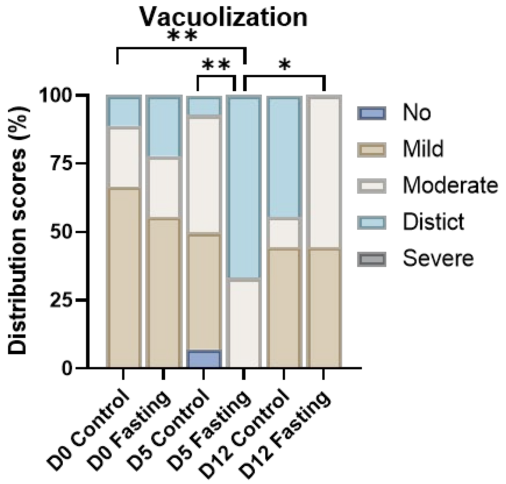
Vacuolization
**
**
*
Distribution scores (%)
100
75
50
25
0
D0 Control
D0 Fasting
D5 Control
D5 Fasting
D12 Control
D12 Fasting
No
Mild
Moderate
Distict
Severe

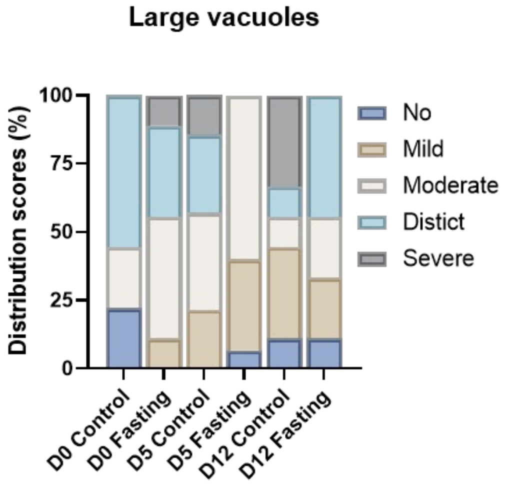
Large vacuoles
Distribution scores (%)
100
75
50
25
0
D0 Control
D0 Fasting
D5 Control
D5 Fasting
D12 Control
D12 Fasting
No
Mild
Moderate
Distict
Severe

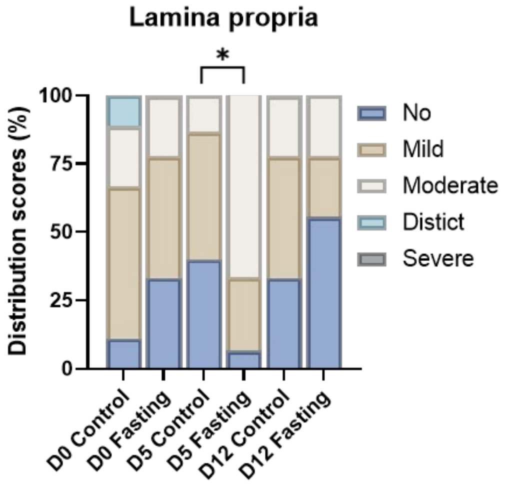
Lamina propria
*
Distribution scores (%)
100
75
50
25
0
D0 Control
D0 Fasting
D5 Control
D5 Fasting
D12 Control
D12 Fasting
No
Mild
Moderate
Distict
Severe

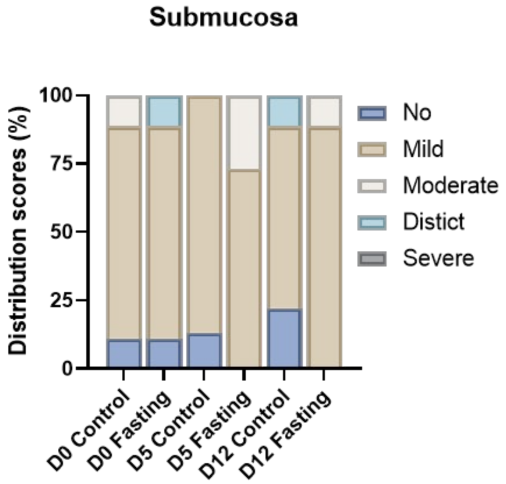
Submucosa
Distribution scores (%)
100
75
50
25
0
D0 Control
D0 Fasting
D5 Control
D5 Fasting
D12 Control
D12 Fasting
No
Mild
Moderate
Distict
Severe

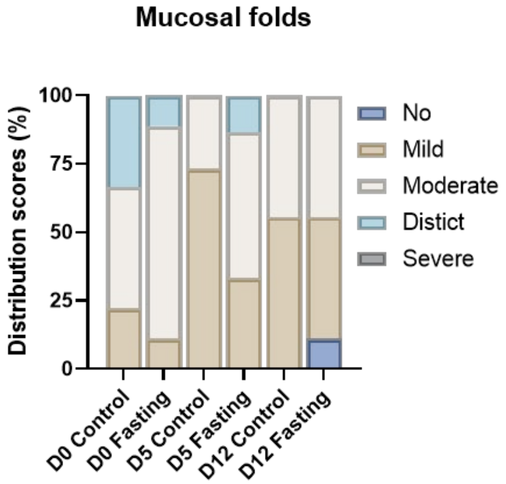
Mucosal folds
Distribution scores (%)
100
75
50
25
0
D0 Control
D0 Fasting
D5 Control
D5 Fasting
D12 Control
D12 Fasting
No
Mild
Moderate
Distict
Severe

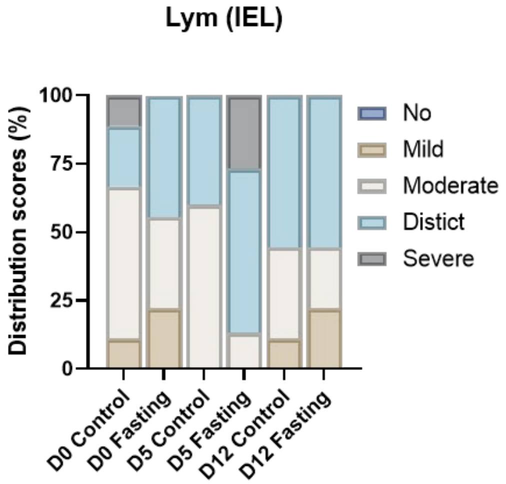
Lym (IEL)
Distribution scores (%)
100
75
50
25
0
D0 Control
D0 Fasting
D5 Control
D5 Fasting
D12 Control
D12 Fasting
No
Mild
Moderate
Distict
Severe

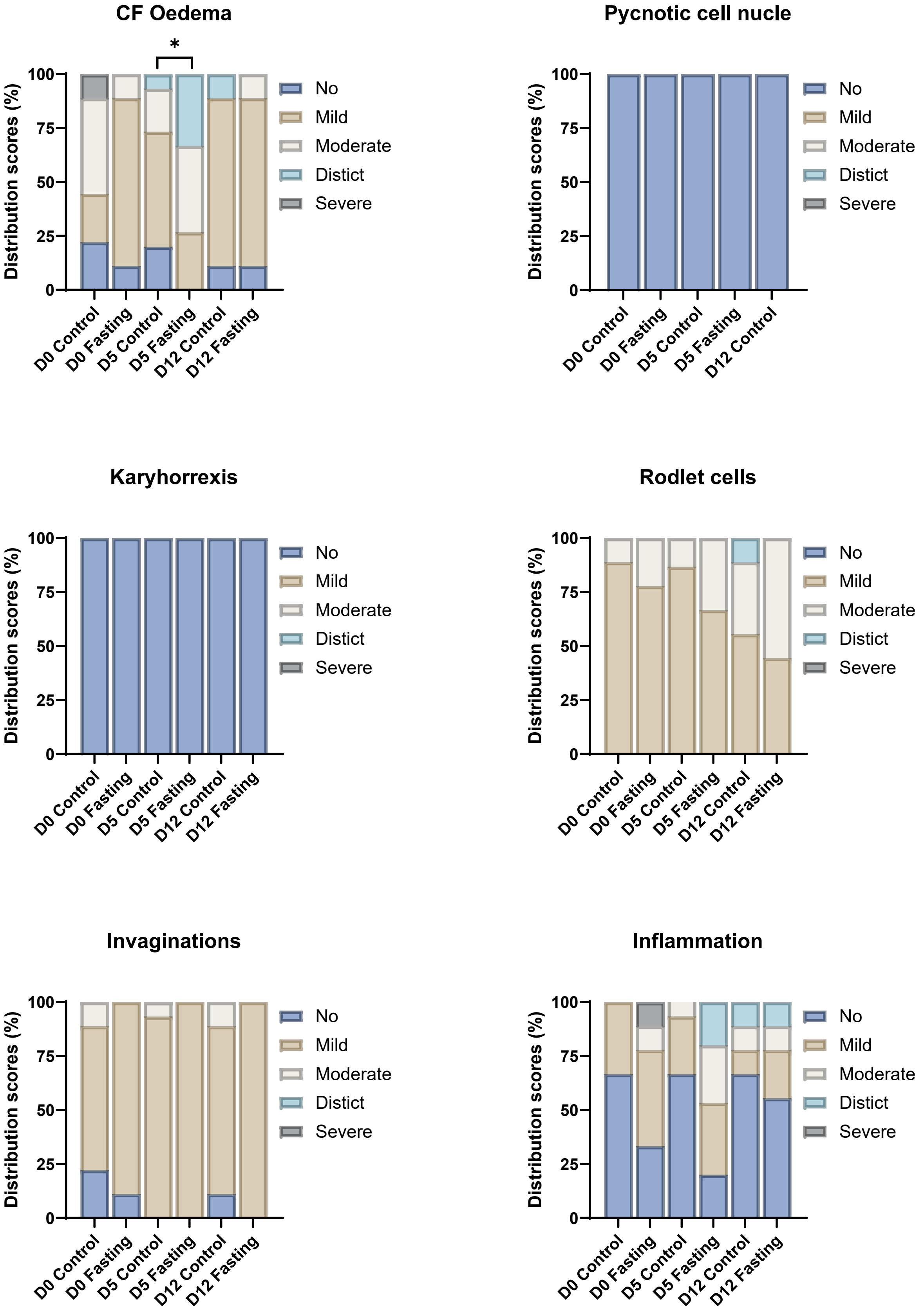
CF Oedema
Pycnotic cell nucle
Karyhorrexis
Rodlet cells
Invaginations
Inflammation
*
Distribution scores (%)
0
25
50
75
100
No
Mild
Moderate
Distict
Severe
D0 Control
D0 Fasting
D5 Control
D5 Fasting
D12 Control
D12 Fasting

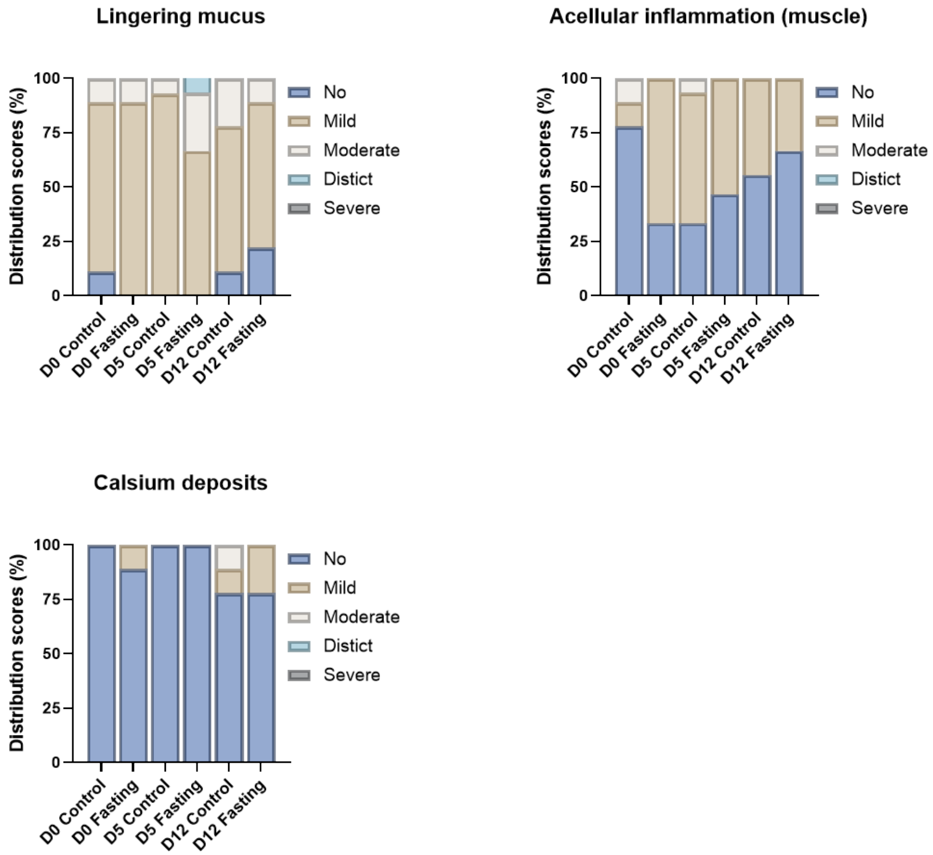

Figure S4 The Atlantic salmon distal intestinal tissue score distribution. The distal intestinal tissue was manually evaluated for 15 characteristic morphological structures. The studied categories were: vacuolization of the enterocytes, large vacuoles in the enterocytes, the thickness of lamina propria (LP) and submucosa (SM), the length and complexity of mucosal folds, interepithelial lymphocytes (IEL) levels, complex fold oedema, pycnotic cell nuclei, karyhorrexis, rodlet cells, mucosal fold invagination, inflammation, lingering mucous, acellular inflammation in muscularis, and calcium deposits. Evaluation was made blindly from n = 9 at D0 and D12, n = 15 at D5 per group. Significant differences between groups were determined by pairwise testing, performed using Kruskal-Wallis and Dunn´s multiple comparison tests: *$p<0.05$, **$p<0.01$.

Table S1 Water quality parameters (mean ± SD) measured of the makeup freshwater (FW) and makeup seawater (SW) for the RAS systems during the experimental period between D-1 and D11

Parameter	Makeup FW mean ± SD	Makeup SW mean ± SD
DO (%)	92.67 ± 2.08	89.67 ± 0.58
DO (mg/L)	10.13 ± 0.47	9.40 ± 0.72
Temperature	7.07 ± 0.81	8.67 ± 0.55
pH	6.84 ± 0.04	8.04 ± 0.03
Conductivity (mS/cm)	0.37 ± 0.06	51.10 ± 0.36
Salinity	0.07 ± 0.06	32.53 ± 0.25
TSS (mg/L)	0.03 ± 0.13	2.74 ± 2.38
Turbidity (NTU)	0.10 ± 0.00	0.10 ± 0.00
Alkalinity (mg $CaCO_3$/L)	15.13 ± 1.15	80.67 ± 5.77
TIC	3.23 ± 0.15	22.63 ± 0.42
TAN	0.10 ± 0.00	0.77 ± 0.81
NO_2N	0.10 ± 0.00	0.10 ± 0.00
NO_3N	0.83 ± 0.38	0.73 ± 0.38

Table S2. Fish measurements from the Control and Fasted RAS groups are presented as the mean of 5 sampled fish per tank (n = 3 tanks; 15 fish per group). Abbreviations GSI = Gonad somatic index, HIS = Heart somatic index, HC = Haematocrit

Variable	Sampling day	Control mean ± SD	Fasted mean ± SD
Weight	D0	135.69 ± 26.34	136.60 ± 28.17
	D5	144.59 ± 24.41	122.20 ± 16.34
	D12	152.75 ± 27.57	161.02 ± 21.43
Length	D0	22.21 ± 1.21	22.16 ± 1.25
	D5	23.01 ± 1.40	22.06 ± 0.97
	D12	23.45 ± 1.13	23.59 ± 1.11
K-factor	D0	1.22 ± 0.07	1.24 ± 0.10
	D5	1.18 ± 0.07	1.13 ± 0.03
	D12	1.18 ± 0.09	1.22 ± 0.05
Gonads	D0	0.06 ± 0.04	0.09 ± 0.05
	D5	0.10 ± 0.05	0.07 ± 0.05
	D12	0.11 ± 0.06	0.11 ± 0.06
Liver	D0	1.31 ± 0.43	1.33 ± 0.37
	D5	1.50 ± 0.31	1.00 ± 0.18
	D12	1.83 ± 0.74	1.88 ± 0.60
GSI	D0	0.04 ± 0.03	0.07 ± 0.04
	D5	0.07 ± 0.04	0.06 ± 0.04
	D12	0.07 ± 0.04	0.07 ± 0.04
HSI	D0	0.96 ± 0.18	0.96 ± 0.10
	D5	1.05 ± 0.21	0.82 ± 0.06
	D12	1.20 ± 0.43	1.17 ± 0.34
HC1%	D0	32.71 ± 3.47	33.40 ± 3.81
	D5	29.60 ± 2.95	31.67 ± 3.60
	D12	31.29 ± 2.92	31.33 ± 3.31
HC2%	D0	33.20 ± 3.32	34.20 ± 3.59
	D5	30.58 ± 2.75	32.42 ± 4.12
	D12	30.53 ± 3.40	30.57 ± 2.98
